# Syndromes microdélétionnels (syndrome de Williams et syndrome de la délétion 22q11) au CHU Hassan II de Fès: à propos de 3 observations

**Published:** 2012-01-12

**Authors:** Karim Ouldim, Laila Bouguenouch, Imane Samri, Ihsan El Otmani, Hasna Hamdaoui, Sanae Bennis, Mounia Idrissi Lakhdar, Sana Chaouki, Samir Atmani, Moustapha Hida

**Affiliations:** 1 Unité de Génétique Médicale et d’oncogénétique, Laboratoire centrale d’analyses Médicales, CHU Hassan II, Fès, Maroc; 2 Service de Pédiatrie CHU Hassan II, Fès, Maroc

**Keywords:** Syndromes microdélétionnels, FISH, Syndrome de Williams, 22q11

## Abstract

Les syndromes microdélétionnels sont définis par la présence d’une anomalie chromosomique de taille mineure (inférieure à 5 mégabases) ou aneusomie segmentaire, décelable par cytogénétique moléculaire (FISH : Fluorescent in Situ Hybridization). Les syndromes microdélétionnels représentent des syndromes cliniques avec des phénotypes suffisamment caractéristiques pour être reconnus cliniquement. Actuellement la FISH est la technique de choix pour rechercher ces syndromes. Plusieurs syndromes microdélétionnels peuvent être confirmés aisément, les plus recherchés sont Le syndrome de Williams (microdélétion en 7q11.23) et le syndrome de la délétion 22q11 (microdélétion en 22q11.2). Le syndrome de Williams est caractérisé par une anomalie du développement qui associe un retard psycho-moteur, une dysmorphie du visage évocatrice et un profil cognitif et comportemental spécifique, une sténose aortique supravalvulaire -SASV- le plus souvent. Le Syndrome de la délétion 22q11 se caractérise par l’association de plusieurs malformations d’expression variable: une cardiopathie congénitale de type conotroncal, une dysmorphie faciale discrète mais caractéristique et une hypoplasie du thymus et des parathyroïdes. Nous rapportons nos premières observations au CHU Hassan II confirmées par FISH : Syndrome de la délétion 22q11 (n:2) et un syndrome de Williams. Le but de cet article est la mise à jour de nos connaissances sur ces deux syndromes et la mise en valeur du rôle de la cytogénétique moléculaire dans le diagnostic et le conseil génétique des syndromes microdélétionnels.

## Introduction

Les syndromes microdélétionnels représentent des syndromes cliniques, associant généralement : un retard mental, une dysmorphie avec des malformations d’organes et des troubles du comportement. Ils sont liés à une microdélétion particulière, non visible sur un caryotype standard. Il s’agit d’un syndrome de gènes contigus le plus souvent de novo par perte de fragment chromosomique de petites tailles infra-microscopiques (< 5 mégabases). Cette microdélétion est décelable uniquement par l′utilisation des techniques de haute résolution ou de cytogénétique moléculaire dont la plus utilisée en pratique médicale est l’Hybridation in situ fluorescente FISH (Fluorescent in Situ Hybridization), qui repère une séquence spécifique par une sonde complémentaire d’ADN marqué en fluorescence [1,2]. Ces syndromes apparaissent lors de certains crossing-over, le misappariement de séquences d’ADN ayant une forte homologie de séquence entraîne la délétion de la zone chromosomique comprise entre les séquences homologues [1]. Les deux syndromes les plus étudiés, diagnostiqués et suivis en pratique médicale sont : le syndrome de la délétion 22_q_11 et le syndrome de Williams. A travers nos trois observations colligées et diagnostiquées et suivies dans notre institution, nous mettrons à jour les dernières actualités scientifiques et en valeur l’intérêt d’une approche multidisciplinaire.

## Observations

### Observation 1: (Figure 1)

Fille de neuf ans, adressée en consultation de génétique médicale et qui présente une légère dysmorphie faciale (Racine du nez aplatie avec extrémité bulbeuse, grande bouche avec lèvre inferieure large et éversée et un long philtrum), un retard psycho moteur, un retard staturo-pondéral, une hypertension artérielle (PAS : 16 mmHg, PAD :11 mmHg). Elle présente aussi un profil cognitif et comportemental, particulier regroupant une : Hypersociabilité, hypersensibilité au bruit, des dispositions pour la musique et un retard cognitif modéré avec un langage préservé. Des troubles du sommeil, des vomissements chroniques avec constipation. L’échocardiographie montre une hypertrophie ventriculaire gauche (Complication d’une HTA sans sténose de l’artère rénale). L’échographie abdomino-rénale et le bilan biologique (numération formule sanguine, ionogramme) sont sans particularités. Les parents sont phénotypiquement normaux.

**Figure 1:**
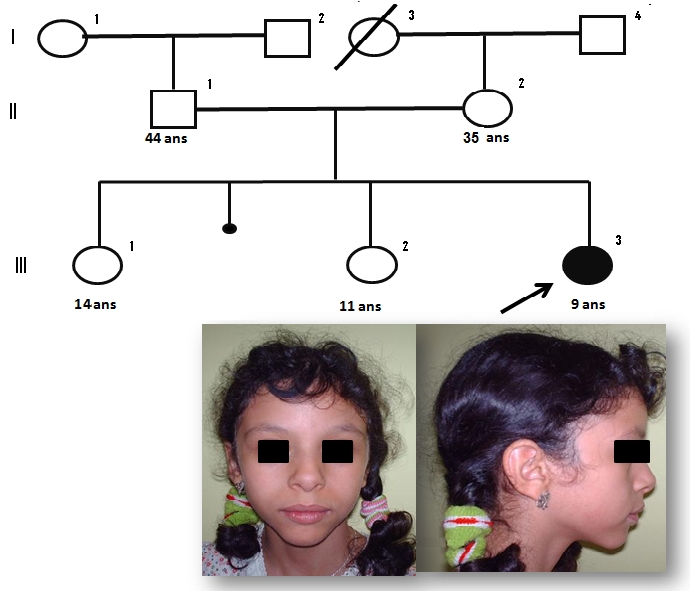
Syndromes microdélétionnels (syndrome de Williams et syndrome de la délétion 22q11) au CHU Hassan II de Fès. Arbre généalogique et aspect facial de l’observation 1

### Observation 2: (Figure 2)

Nourrisson de 12 mois, de sexe féminin adressée en consuultation de génétique médicale et qui présente une légère dysmorphie faciale : nez tubulaire, des arêtes nasales larges et proéminentes et des narines antéversées, des oreilles petites, mal ourlées, décollées et bas implantées, un philtrum court, une cardiopathie congénitale de type conotroncal (tétralogie de Fallot, atrésie pulmonaire), une fente palatine, un retard de croissance et mauvaise prise pondérale, une hypotonie musculaire et une hernie ombilicale. L’échographie abdomino-rénale, thymique et l’électro-encéphalogramme sont normaux. Le bilan immunologique, la calcémie, la numération formule sanguine, l’ionogramme ne montrent aucune anomalie. Les parents sont de phénotype normal.

**Figure 2:**
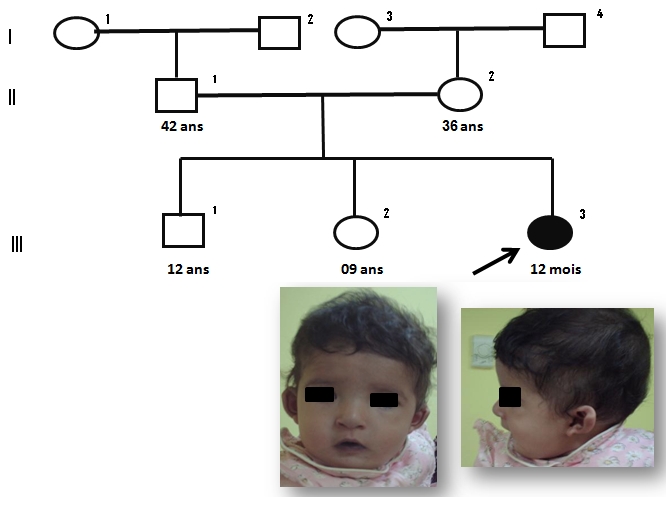
Syndromes microdélétionnels (syndrome de Williams et syndrome de la délétion 22q11) au CHU Hassan II de Fès. Arbre généalogique et aspect facial de l’observation 2

### Observation 3: (Figure 3)

Nourrisson de 3 mois, de sexe féminin, qui présente une légère dysmorphie faciale, une tétralogie de Fallot, associée à une atrésie pulmonaire et CIV, un retard de croissance et mauvaise prise pondérale, une hypotonie musculaire, héxadactylie des doigts post axial bilatérale. L’échographie abdomino-rénale, thymique et l’électro-encéphalogramme étaient normaux. Le bilan immunologique et la calcémie étaient par ailleurs normaux. Les parents sont de phénotype normal.

**Figure 3:**
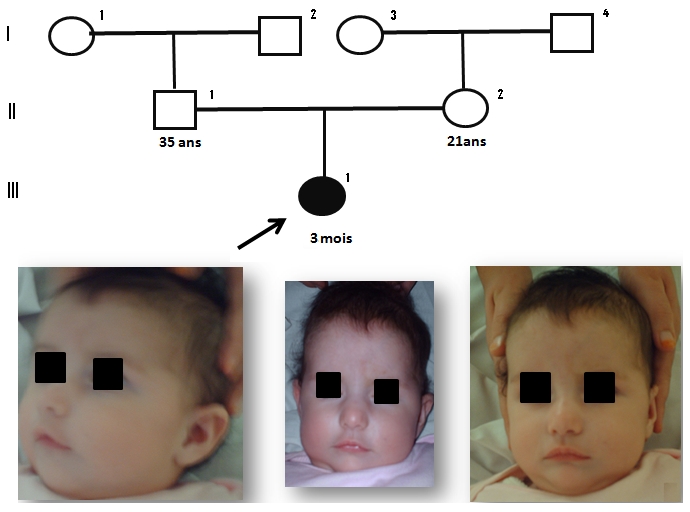
Syndromes microdélétionnels (syndrome de Williams et syndrome de la délétion 22q11) au CHU Hassan II de Fès. Arbre généalogique et aspect facial de l’observation 3

### Méthodes utilisées

Devant ces tableaux cliniques, nous avons suspecté une microdélétion 7q11.23 pour l’observation 1 et une microdélétion 22q11.2 pour les observations 2 et 3. Les préparations chromosomiques sont obtenues à partir de la culture des lymphocytes sanguins selon la technique classique avec étalement sur des lames. Un caryotype constitutionnel métaphasique en Bandes R, à la recherche d’une éventuelle translocation impliquant les chromosomes 7 pour l’observation 1 et les chromosomes 22 pour les observations 2 et 3 et une hybridation in situ fluorescente sur préparations chromosomiques (FISH : Fluorescence In Situ Hybridization) ont été effectués. Les sondes utilisées sont (FISH sur chromosomes métaphasiques et noyaux) : Pour l’observation 1: Vysis Williams Region Probe - LSI ELN SpectrumOrange/LSI D7S486, D7S522 SpectrumGreen. Pour l’observation 2 et 3 (propositus et parents): Vysis DiGeorge Region Probe- LSI TUPLE 1 SpectrumOrange 22q11.2 /LSI ARSA SpectrumGreen 22q13.

**Figure 4:**
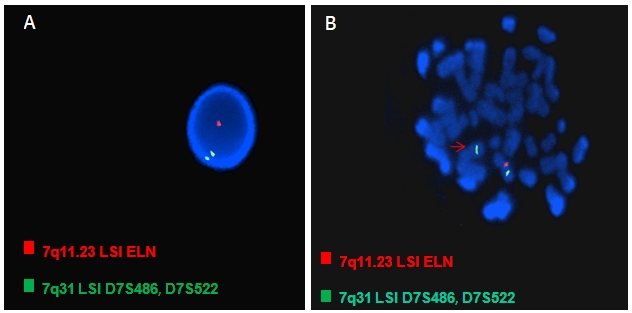
Présence d’une microdélétion hémizygote en position 7q11.23: Syndrome de Williams. (A) Présence d’un spote rouge et deux spotes vert sur noyaux colorés par le DAPI. (B) la flèche montre l’absence du spote rouge sur un de deux chromosomes 7

**Figure 5:**
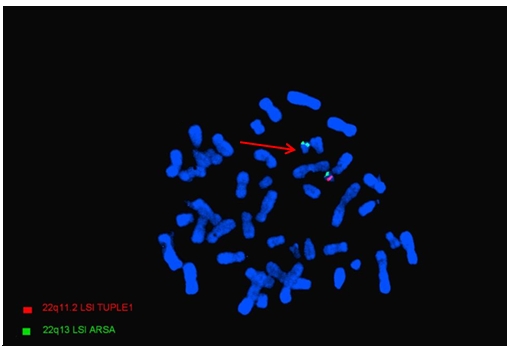
Présence d’une microdélétion hémizygote en position 22q11.2 : Syndrome de délétion 22q11 (la flèche montre l’absence du spote rouge sur un de deux chromosomes 22)

Nous avons confirmé par FISH le diagnostic du Syndrome de Williams pour l’observation 1 : une microdélétion chromosomique hémizygote en position 7q11.23 (Figure 4) et le Syndrome de la délétion 22q11 pour l’observation 2 et 3 : microdélétion hémizygote en position 22q11.2 (Figure 5). La FISH sur préparation chromosomique des parents de l’observation 2 et 3 ne montrent aucune délétion, ce qui confirme que la délétion 22q11 chez les deux propositus de l’observation 2 et 3 est de *novo*. Pas d’indication médicale pour explorer les parents de phénotype normal de l’observation 1. Les détails des résultats et les formules cytogénétiques des différentes observations sont résumés dans le tableau 1.

**Tableau 1:** **Résultats** de la cytogénétique classique et moléculaire chez trois patients avec Syndromes microdélétionnels (syndrome de Williams et syndrome de la délétion 22q11) au CHU Hassan II de Fès

Observation	Résultats cytogénétique	Diagnostic
Observation 1 (Figure 4)	46,XX.ish del(7)(q11.23q11.23)(ELN-)[11] (Figure 4b) nuc ish(ELN×1)(D7S522×2)[100] (Figure 4 (A))	Syndrome de Williams
Observation 2 (Figure 5)	46,XX.ish del(22)(q11.2q11.2)(TUPLE1-)[10]nuc ish(TUPLE1×1)(ARSA×2)[100]	Syndrome de la délétion 22q11
Observation 2	46,XX.ish del(22)(q11.2q11.2)(TUPLE1-)[10]nuc ish(TUPLE1×1)(ARSA×2)[100]	Syndrome de la délétion 22q11

## Discussion

L′apparition des techniques de haute résolution chromosomique dans les années 1980 a permis de mettre en évidence des microremaniements dans des syndromes individualisés dans les années 60. Les syndromes microdélétionnels sont des pathologies génétiques rares, ils ont été définis comme la présence de perte de fragments chromosomiques de petites tailles (< 5 mégabases) dont le phénotype est constitué d’une association de signes caractéristiques décelable uniquement par l′utilisation des techniques de haute résolution ou de cytogénétique moléculaire (Hybridation in situ fluorescente, FISH). Le plus souvent il s’agit d’une délétion de novo c’est-à-dire non transmise par les parents mais liée à un accident méiotique [4].

La cytogénétique moléculaire d’apparition relativement récente est une discipline frontière entre la cytogénétique et la génétique moléculaire qui a révolutionné′ l’approche traditionnelle de la cytogénétique. Actuellement, ses outils principaux sont l’hybridation in situ en fluorescence (FISH) sur préparation chromosomique et la comparative genomic hybridization-array (CGH-array). Le principe de la FISH repose sur l’hybridation moléculaire : une sonde spécifique de la région à explorer et marquée par un fluorochrome peut s’hybrider, grâce à la complémentarité des bases nucléotidiques, spécifiquement avec sa séquence-cible sur une préparation de noyaux en interphase ou en métaphase. Les sondes locus spécifiques sont construites pour explorer spécifiquement une région chromosomique donnée. La FISH permet de caractériser des microremaniements non détectables sur un caryotype standard mais évoques devant un tableau clinique particulier, c’est le cas des syndromes microdélétionnels, les plus étudiés et fréquemment diagnostiqués en pratique médicale, c’est sans doute le syndrome de la délétion 22q1 et le syndrome de Williams-Beuren (SWB) [1]. Le syndrome de Williams-Beuren (SWB) est une maladie rare puisque sa prévalence à la naissance a pu être évaluée entre 1/7500 et 1/20 000 [5–7]. Il est dû à une microdélétion chromosomique en 7q11.23 englobant 28 gènes dont celui de l’élastine (ELN), composant essentiel du tissu extracellulaire artériel [2].

Les anomalies du développement associent classiquement une dysmorphie du visage assez spécifique, des malformations cardiovasculaires (le plus souvent une sténose aortique supravalvulaire ou une sténose des branches de l’artère pulmonaire) et un profil neuropsychologique spécifique [5–8]. Ce profil neuropsychologique se caractérise principalement par un retard cognitif modéré, un langage relativement préservé, des déficits visuospatiaux et une hypersociabilité [9,10]. D’autres manifestations moins connues et moins fréquentes peuvent bénéficier d’une prise en charge, telles que l’hypercalcémie néonatale, des troubles digestifs de la petite enfance, des problèmes ophtalmologiques, l’hypothyroïdie, un retard de croissance, des manifestations articulaires, des anomalies buccodentaires et une hypertension apparaissant à l’adolescence ou à l’âge adulte. Ce syndrome est de transmission autosomique dominante, avec un risque de récurrence de 50% pour les patients atteints, La microdélétion impliquée survient presque toujours de novo, d’où le caractère habituellement sporadique de cette affection [11]. La survenue préférentielle et récurrente de la même délétion chez des individus différents est due à la présence dans cette région de 2 séquences low copy repeats (LCR). Ces séquences d’ADN à très forte homologie sont à l’origine, au cours de la méiose, de recombinaisons homologues non alléliques, c’est-a-dire de défauts d’appariement conduisant à la formation de gamètes contenant un chromosome 7 présentant soit une délétion, soit une duplication réciproque de la région 7q11.23 [12].

Notre patiente présente un phénotype spécifique, un profil comportemental et cognitif et une hypertension artérielle rapportée dans le syndrome de Williams. Nous avons confirmé par FISH la microdélétion par FISH sur métaphase et noyaux. Une prise en charge multidisciplinaire est mise en route (pédiatre, cardiopédiatre, orthodontiste, orthopédiste, ophtalmologiste, etc.). Dans le cas du notre couple, phénotypiquement normal, il n’y pas d’indication d’explorer les parents par FISH, la survenue de ce syndrome s’explique par une microdélétion de novo (il n’y a jamais eu de cas rapporté de microdélétion en 7q11.23 avec un phénotype normal [12]). Le risque de récurrence d’un Syndrome de williams chez notre couple est très faible mais non nul, lié au mosaïcisme germinale. Le syndrome de la délétion 22q11.2 (del22q11) est l’un des syndromes neurogénétiques le plus fréquent et résulte dans la majorité des cas, d’une microdélétion de novo sur le chromosome 22. La prévalence de la microdélétion 22 est estimée à environ 1/4000-6000 naissances, ce qui en fait l’un des syndromes neurogénétiques les plus courants [3]. Le syndrome de la délétion 22q11.2 (del22q11) résulte généralement d’une microdélétion de novo d’environ 3Mb sur le bras long (q) du chromosome 22 en position 22q11.2. Les formes familiales surviennent dans environ 10% des cas et sont transmises sur le mode autosomique dominant [3,13]. Ces dernières pourraient engendrer des manifestations cliniques de plus grande sévérité. Le syndrome de la del22q11 implique plus de 35 gènes et possède un phénotype très hétérogène, tant sur le plan physique que cognitif ou comportemental [14] Le syndrome de la délétion 22q11.2 possède un phénotype étendu et variable. En effet, plus de 180 caractéristiques cliniques ont été recensées dans ce syndrome [14–16] Les microdélétions 22q11.2 sont mises en évidence chez des patients pouvant présenter un phénotype différent : syndrome de Di George (99% des cas), syndrome vélocardiofacial (70% des cas), le conotruncal anomaly face syndrome, le caylor cardiofacial syndrome et le syndrome autosomique dominant d’Opitz ou encore des personnes avec un phénotype très modéré (10% des parents). La dysmorphie faciale est quasi constante mais difficile à diagnostiquer car elle est le plus souvent discrète. Elle comprend un nez tubulaire, saillant à la racine courte avec des arêtes nasales larges et proéminentes, et des narines antéversées. Les oreilles sont petites, mal ourlées, décollées et bas implantées. Le phénotype peut se compléter par une microstomie avec un philtrum court et des fentes palpébrales droites [17]. Certains signes cliniques de ce syndrome sont découverts en période néonatale, en particulier les malformations cardiaques, l’hypoplasie thymique et l’hypocalcémie néonatale [18]. La cardiopathie est la malformation la plus fréquente (75%), elle est de type conotroncale : interruption de l’arc aortique, troncus arteriosis, tétralogie de Fallot. Bonnet et al. ont rapporté que 50% des malformations conotroncales étaient associées au syndrome de Di George [19]. Des anomalies otolaryngées, rénales et paratyroïdiennes ainsi qu’une hypotonie sont également fréquemment associées [14]. De la même manière que le phénotype physique est extrêmement variable d’un individu à l’autre, le profil cognitif des personnes porteuses de la del22q11 est également hétérogène. Si un peu moins de la moitié des enfants et adolescents présentent un retard du développement intellectuel (QI<70), la majorité ont des troubles des apprentissages [20]. Plus de 85% des malades présentent une microdélétion 22q11.2 d’environ 3 millions de nucléotides. Cette grande région chromosomique contient de nombreux gènes, dont le gène TBX1, qui jouerait un rôle important dans le phénotype des microdélétions 22q11.2. Cette microdélétion 22q11.2 doit être recherchée par la technique d’hybridation in situ en fluorescence (FISH) [14,21]. La plupart des microdélétions sont de novo (93% des cas), alors que la microdélétion n’est héritée d’un des parents selon un mode de transmission autosomique dominant, que dans 7% des cas [22]. La mosaïque germinale (récurrence alors que les parents ne sont pas porteurs de la microdélétion) est exceptionnelle. Les patients ayant une microdélétion 22q11.2 ont 50% de risque de la transmettre à leur descendance. La grande variabilité inter et intra familiale rend toute corrélation génotype-phénotype difficile, d’où la nécessité d’explorer par FISH systématiquement les parents avec phénotype normal d’un enfant porteur d’une délétion 22q11 [23].

Le pronostic vital est essentiellement conditionné par la sévérité de la cardiopathie congénitale. À long terme, le pronostic dépend de l’association d’un retard mental et des troubles du comportement. La prise en charge d’un enfant porteur d’une microdélétion 22q11.2 est variable et dépend avant tout des malformations et complications associées (cardiopathie, hypocalcémie…) [24]

Nous avons confirmé par FISH la microdélétion 22q11.2 chez nos deux patients de l’observation 2 et 3 qui présentaient un phénotype évoquant le syndrome de la délétion 22q11. Le conseil génétique est rassurant puisque l’exploration par FISH de la microdélétion 22q11 sur préparations chromosomiques des parents de l’observation 2 et 3 ne montre pas de microdélétion en position 22q11. Le risque de récurrence chez nos deux couples du syndrome de la délétion 22q11 est très faible mais non nul, lié au mosaïcisme germinale. Une prise en charge multidisciplinaire est mise en route selon les dernières recommandations du consortium international du syndrome de la délétion 22q11.2 [24].

## Conclusion

L’apport de cytogénétique moléculaire (FISH) est capital pour l’exploration des syndromes microdélétionnels ayant pour but la confirmation diagnostic, le conseil génétique et la prise en charge qui est le plus souvent multidisciplinaire. Les syndromes les mieux étudiés et plus explorés en pratique médicale sont le syndrome de Williams (microdélétion en 7q11.23) et le syndrome de la délétion 22q11 (microdélétion en 22q11.2). A travers cet article nous mettons en valeur le rôle des explorations cytogénétiques classiques et moléculaires dans la prise en charge de l’handicap d’origine génétique pour une meilleure prise en charge et un conseil génétique adéquat des patients et de leurs familles. La CGH-array (Comparative Genomic Hybridization-array) (puces ADN) est une méthode d’exploration pangénomique, qui représente un moyen plus sensible que la FISH, plus rapide pour l’exploration des microremaniements (microdélétions, microduplications) génomiques jusqu’à maintenant non diagnostiquées. Néanmoins le coût du CGH-array et de sa plateforme technique ainsi que les difficultés d’interprétation des résultats représentent actuellement un handicap pour sa généralisation en pratique clinique dans le future proche.
